# Exploring the efficacy of tumor electric field therapy against glioblastoma: An *in vivo* and *in vitro* study

**DOI:** 10.1111/cns.13750

**Published:** 2021-10-28

**Authors:** Hao Wu, Lin Yang, Hanjie Liu, Dan Zhou, Dikang Chen, Xiaoque Zheng, Hui Yang, Chong Li, Jiusheng Chang, Anhua Wu, Zhifei Wang, Nianjun Ren, Shengqing Lv, Yuyang Liu, Muyuan Jia, Jian Lu, Hongyu Liu, Guochen Sun, Zhixiong Liu, Jialin Liu, Ling Chen

**Affiliations:** ^1^ Department of Neurosurgery, The Third Xiangya Hospital Central South University Changsha China; ^2^ Department of Neurosurgery The First Medical Center Chinese PLA General Hospital Beijing China; ^3^ Beijing Neurosurgical Institute; Department of Neurosurgery, Beijing Tiantan Hospital Capital Medical University Beijing China; ^4^ Hunan An Tai Kang Cheng Biotechnology Co., Ltd Changsha China; ^5^ Department of Anesthesiology, The Third Xiangya Hospital Central South University Changsha China; ^6^ Institute of Biophysics Chinese Academy of Sciences Beijing China; ^7^ Department of Neurosurgery The First Hospital of China Medical University Shenyang China; ^8^ Department of Neurosurgery, The Affiliated Cancer Hospital of Xiangya School of Medicine Central South University/Hunan Cancer Hospital Changsha China; ^9^ Department of Neurosurgery Xinqiao Hospital, Third Military Medical University Chongqing China; ^10^ Department of Neurosurgery Xiangya Hospital, Central South University Changsha China

**Keywords:** glioblastoma, individualized therapy, primary tumor cells, random sequence output mode, tumor electric field therapy system

## Abstract

**Aims:**

Tumor electric fields therapy (TTFields) is emerging as a novel anti‐cancer physiotherapy. Despite recent breakthroughs of TTFields in glioma treatment, the average survival time for glioblastoma patients with TTFields is <2 years, even when used in conjugation with traditional anti‐cancer therapies. To optimize TTFields‐afforded efficacy against glioblastoma, we investigated the cancer cell‐killing effects of various TTFields paradigms using *in vitro* and *in vivo* models of glioblastoma.

**Methods:**

For *in vitro* studies, the U251 glioma cell line or primary cell cultures prepared from 20 glioblastoma patients were treated with the tumor electric field treatment (TEFT) system. Cell number, volume, and proliferation were measured after TEFT at different frequencies (100, 150, 180, 200, or 220 kHz), durations (24, 48, or 72 h), field strengths (1.0, 1.5, or 2.2V/cm), and output modes (fixed or random sequence output). A transwell system was used to evaluate the influence of TEFT on the invasiveness of primary glioblastoma cells. For *in vivo* studies, the therapeutic effect and safety profiles of random sequence electric field therapy in glioblastoma‐transplanted rats were assessed by calculating tumor size and survival time and evaluating peripheral immunobiological and blood parameters, respectively.

**Results:**

In the *in vitro* settings, TEFT was robustly effective in suppressing cell proliferation of both the U251 glioma cell line and primary glioblastoma cell cultures. The anti‐proliferation effects of TEFT were frequency‐ and “dose” (field strength and duration)‐dependent, and contingent on the field sequence output mode, with the random sequence mode (TEFT‐R) being more effective than the fixed sequence mode (TEFT‐F). Genetic tests were performed in 11 of 20 primary glioblastoma cultures, and 6 different genetic traits were identified them. However, TEFT exhibited comparable anti‐proliferation effects in all primary cultures regardless of their genetic traits. TEFT also inhibited the invasiveness of primary glioblastoma cells in transwell experiments. In the *in vivo* rat model of glioblastoma brain transplantation, treatment with TEFT‐F or TEFT‐R at frequency of 200 kHz and field strength of 2.2V/cm for 14 days significantly reduced tumor volume by 42.63% (TEFT‐F vs. control, *p* = 0.0002) and 63.60% (TEFT‐R vs. control, *p* < 0.0001), and prolonged animal survival time by 30.15% (TEFT‐F vs. control, *p* = 0.0415) and 69.85% (TEFT‐R vs. control, *p* = 0.0064), respectively. The tumor‐bearing rats appeared to be well tolerable to TEFT therapies, showing only moderate increases in blood levels of creatine and red blood cells. Adverse skin reactions were common for TEFT‐treated rats; however, skin reactions were curable by local treatment.

**Conclusion:**

Tumor electric field treatment at optimal frequency, strength, and output mode markedly inhibits the cell viability, proliferation, and invasiveness of primary glioblastoma cells *in vitro* independent of different genetic traits of the cells. Moreover, a random sequence electric field output confers considerable anti‐cancer effects against glioblastoma *in vivo*. Thus, TTFields are a promising physiotherapy for glioblastoma and warrants further investigation.

## Graphical abstract

Primary tumor cells prepared from 20 patients were treated with the tumor electric field treatment system (TEFTS). The impact of genetic background on cell responses to TEFTS were evaluated. Cell volume, cell number and cell proliferation were measured after TEFT at different frequencies (100, 150, 180, 200, 220 kHz). A transwell system was used to evaluate the influence of TEFT on the invasiveness of primary cells. The therapeutic effect and safety of random sequence electric field therapy in tumor‐bearing rats were assessed by calculating tumor size and survival time and by evaluating other immunobiological and blood parameters. Twelve types of primary tumor cells with different genetic traits were all sensitive to electric field treatment. After sensitive frequency treatment, the volume of primary cells significantly increased compared to control group, while the proliferation and invasiveness of tumor cells were inhibited. The optimal frequencies among all 20 types of primary cells were divided into different categories: 30% of them had the optimal frequency of 180 kHz; 25% of them had the optimal frequency of 200 kHz; 15% of them had the optimal frequency of 150 kHz, and 30% of them had the optimal frequency of 220 kHz. Two cases were not sensitive to 200 kHz. The therapy with random sequence electric field output significantly reduced tumor size and elongated survival time of tumor‐bearing rats compared to the fixed sequence TEFT, while increased the incidence of dermatological side effects.

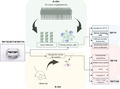

## 1 INTRODUCTION

In 2004, KIRSON et al.^1^ report for the first time in a variety of tumor cell lines and in animal models of malignant tumors that alternating electric fields of intermediate frequency (100–300 kHz) and low intensity (1‐3V/cm) exert antimitotic effects selectively to dividing tumor cells, while have no effect on quiescent cells. Since then, the tumor electric fields or tumor‐treating fields (TTFields) have been brought to the attention of more and more basic scientists and clinicians.^2^, ^3^, ^4^, ^5^, ^6^

The TTFields interfere with the rapid division of tumor cells by acting on the spindle assembly in the middle and late stages of mitosis. In addition, uneven electric fields generated in mitotic tumor cells affect the organelles and macromolecules in the cells during the division period, resulting in abnormal chromosome segregation, multinucleated cells, and apoptosis.^7^, ^8^, ^9^, ^10^ Recently, breakthroughs have been made in the research on TTFields. Studies have found that it can enhance the sensitivity of tumor cells to DNA‐damaging agents^11^ and increase the permeability of tumor cell membranes.^12^ In addition, low‐frequency TTFields (100 kHz) can change the integrity of the blood‐brain barrier (BBB). ^13^The increased BBB permeability makes it possible to deliver drugs directly to the brain.^13^ The tumor cell damages caused by TTFields promote the immune response.^14^, ^15^, ^16^, ^17^ Along with the advancement in preclinical research, clinical trials of TTFields have also been extensively carried out, including a phase II clinical trial of triple treatment with temozolomide + pembrolizumab,^18^ the phase I study of a combined treatment with a personalized neoantigen vaccine in newly diagnosed glioblastoma (NCT03223103), and a clinical trial of combined treatment with radiotherapy alone or with radiotherapy and temozolomide for newly diagnosed patients (NCT03477110 and NCT03780569). With those advances in treating studies, patients with nervous tumor have potential to improve their prognosis.^19^, ^20^

In short, TTFields are a new type of treatment that utilizes low‐intensity, medium‐frequency, and alternating electric fields to exert biophysical forces on various charged and polarized molecules, resulting in a series of biological effects.^21^ Its efficacy, reliability, and safety depend on specific parameters, including intermediate frequency (100~300 kHz) and low intensity (1~3V/cm).^22^, ^23^ Previous studies have shown that different tumor cell lines are sensitive to different electric field frequencies,^24^ and the optimal frequency is inversely proportional to the size of the tumor cell.^1^ The most suitable parameters for electric field therapy in glioma are frequency at 200 kHz and intensity at 1~2V/cm. Additionally, the efficacy of TTFields relies on the relative direction of the mitotic axis and the field vector. In a single directional electric field, the damage to tumor cells increased 5 times when the mitotic axis was oriented the same as the electric field.^1^ It is unknown whether the polyclonal primary tumor cells from different patients are all sensitive to the parameters above, and whether specific genetic components (such as O6‐methylguanine‐DNA methyltransferase (MGMT), isocitrate dehydrogenase IDH mutations, etc.) respond to TTFields. Less is known about the therapeutic effect of multi‐directional random sequence electric field compared with bi‐directional electric field.

Here, we adopted a domestic TEFT system to evaluate the treatment parameters in the glioma U251 cell line. We found that the domestic system can achieve comparable efficacy when the relative duration, field strength, frequency, and other parameters remain the same as the similar foreign products. We also used domestic systems to treat the cultured primary tumor cells collected from different glioma patients. The changes in cell volume, cell number, cell proliferation, and invasiveness were assessed. Finally, we improved the TEFT system by increasing the direction of the electric field from 1 to 3 random sequential directions. The therapeutic effect and safety of such random sequence electric field output were assessed in tumor‐bearing rats by calculating tumor size and survival time and evaluating other immunobiological and blood parameters. Our results showed that all primary glioma cells with different genetic backgrounds responded to electric field treatment. They were sensitive to wide range of frequencies ranging from 100 to 220 kHz. Most of them had optimal frequencies at 200 kHz, and very few glioma cell types did not respond to 200 kHz TEFT. Three directional random sequence electric field output inhibited tumor growth and increased survival time compared with bi‐directional fixed sequence electric field output. Mechanistically, both fixed and random sequence electric fields inhibited tumor cell proliferation, promoted cell apoptosis, and increased the infiltration of CD8^+^ T cells into the tumor mass. The random sequence electric field output showed better efficacy to inhibit proliferation and promote apoptosis. This study suggests that different glioma patients respond differently to various frequencies of TFET. The clinical application of TEFT should be personalized. Random sequence electric field output can improve the therapeutic effect of TEFT.

## 2 MATERIALS AND METHODS

### 2.1 Cultures of glioblastoma cell line

U251 cell line was purchased from the Institute of Basic Medicine, China Medical College. Cells were cultured in DMEM medium (Dulbecco's Modified Eagle's Medium‐High Glucose, Thermo Scientific Fisher) containing 10% fetal bovine serum (FBS, Thermo fisher) in an incubator at 37°C and 5% CO_2_.

### 2.2 Tumor electric field therapy in cell lines

**FIGURE 1:**
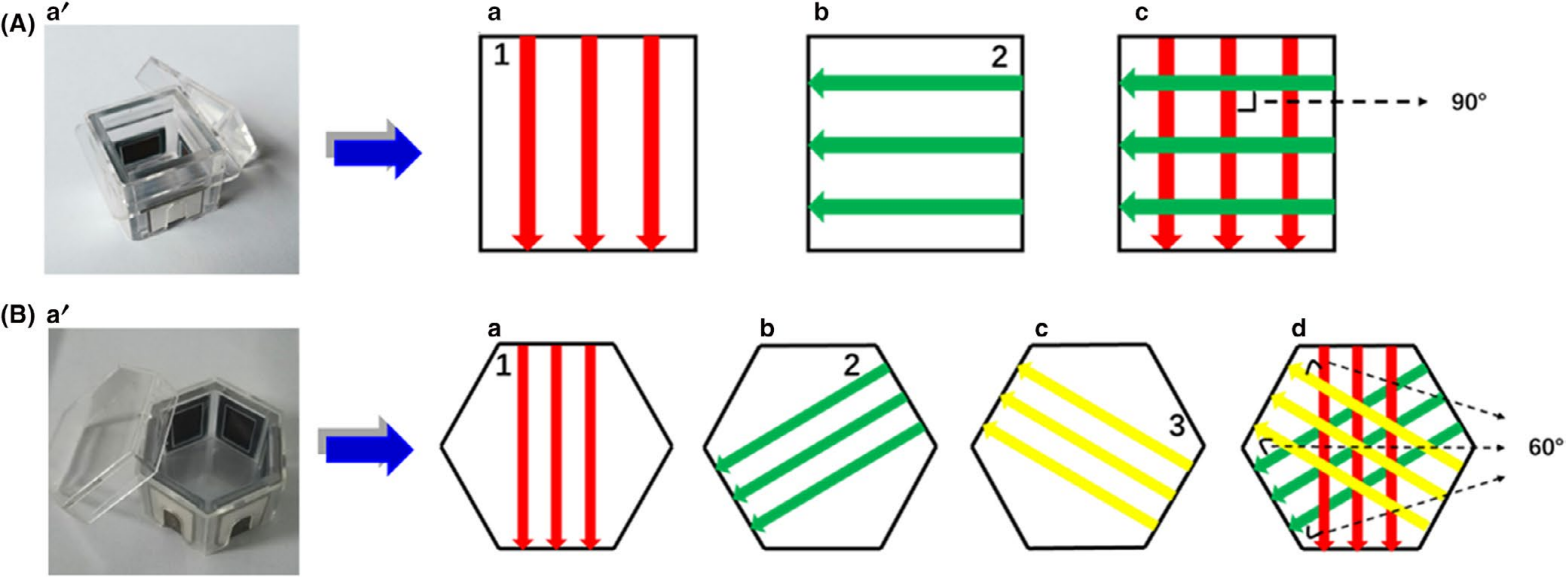
Schematic diagram of the orderly (fixed) and random sequence output modes generated by the tumor electric field treatment system for *in vitro* experiments. (A) When the output of electric field was in an orderly sequence, the cell suspensions were evenly inoculated into a quadrilateral petri dish (a') and electrodes were placed on the four sides of the quadrilateral petri dish. The electric field output of fixed frequency and strength was applied at the directions as illustrated (a, b) and the two directions alternated every second (c). (B) When the output of electric field was in a random sequence, the cell suspensions were inoculated into a hexagonal petri dish (a') and electrodes were placed on the six sides of the hexagonal petri dish. The electric field output was applied at the directions as illustrated (a, b, c), and the three directions were switched randomly every second (d)

The tumor electric field treatment system (TEFTS, CL‐301A) and the special electric field cell culture device were provided by Antai Kangcheng Biotechnology Co., Ltd. The working principles of the random sequence and fixed sequence output modes are shown in Figure 1, and the specific parameter settings are shown in Appendix 1.

A 20 mm diameter glass slide (Nest 801008) was placed in a ceramic petri dish. The tumor electric field intervention device (Antai Kangcheng Biotechnology Co., Ltd. CL‐301A) was installed. 100–150 µL cell suspension (density 2 × 10^5^ cells/mL) was applied evenly on a slide. After a 4–6 h incubation in a 37°C incubator, the medium was supplemented and cells were cultured overnight under the same conditions. The electric field treatment group was subjected to the TEFT with specific parameters (field strength and frequency), and the incubator temperature was set to ensure that the temperature in the petri dish was 37°C. The slides were taken out at specified times, and the cells were digested and counted. The inhibition rate was calculated as the relative number of cells % = (total number of cells in TEFT group/total number of cells in non‐treated group)×100%. The smaller the relative number of cells, the better the inhibitory effect on tumor cells. The experiment was repeated 3 times independently.

### 2.3 Glioma tissue specimen

Human glioma tissues were collected from the Department of Neurosurgery, General Hospital of the Chinese People's Liberation Army. Twenty cases of glioma were surgically resected and pathologically diagnosed as glioma grade III and IV (glioblastoma) according to the WHO pathological classification. The patient was informed of the purpose of this study before the operation. The patient knew the content of the study and signed an informed consent. The study was approved by the ethics review committee of the PLA General Hospital (batch number: S2020–200–02). The clinical data of the patients are shown in Table 1.

**TABLE 1:** Clinical data of 20 glioma patients

NO.	Age	Gender	WHO grade	MGMT status	LOH		IDH−1 (R132H)	IDH−2 (R172)	TERT		BRAF (V600E)	Primary	Recurrent
					1p	19q			C228T	C250T			
T1	67	F	Ⅳ	Non‐methylation	Integrity	Integrity	WT	WT	WT	WT	MT	√	
T2	48	F	Ⅳ	Non‐methylation	Integrity	Integrity	WT	WT	MT	WT	WT	√	
T3	62	F	Ⅳ	Methylation	Integrity	Integrity	WT	WT	MT	WT	WT	√	
T4	70	M	Ⅳ	Methylation	Integrity	Deletion	WT	WT	MT	WT	WT	√	
T5	70	F	Ⅳ	Non‐methylation	Deletion	Integrity	WT	WT	MT	WT	WT	√	
T6	66	F	Ⅳ	Methylation	Integrity	Integrity	WT	WT	MT	WT	WT	√	
T7	56	M	Ⅲ	N/A	N/A	N/A	N/A	N/A	N/A	N/A	N/A	√	
T8	31	M	Ⅳ	Methylation	Integrity	Deletion	MT	WT	WT	WT	WT	√	
T9	62	F	Ⅳ	Methylation	Integrity	Deletion	WT	WT	MT	WT	WT	√	
T10	48	M	Ⅳ	N/A	N/A	N/A	N/A	N/A	N/A	N/A	N/A		√
T11	53	M	Ⅳ	N/A	N/A	N/A	N/A	N/A	N/A	N/A	N/A	√	
T12	66	F	Ⅳ	N/A	N/A	N/A	N/A	N/A	N/A	N/A	N/A	√	
T13	67	M	Ⅳ	Methylation	Integrity	Integrity	WT	WT	WT	MT	WT	√	
T14	55	M	Ⅳ	N/A	N/A	N/A	N/A	N/A	N/A	N/A	N/A	√	
T15	63	F	Ⅳ	N/A	N/A	N/A	N/A	N/A	N/A	N/A	N/A	√	
T16	62	F	Ⅳ	N/A	N/A	N/A	N/A	N/A	N/A	N/A	N/A	√	
T17	46	M	Ⅳ	N/A	N/A	N/A	N/A	N/A	N/A	N/A	N/A	√	
T18	50	M	Ⅳ	Non‐methylation	Integrity	Integrity	WT	WT	WT	WT	WT		√
T19	64	F	Ⅳ	Methylation	Integrity	Integrity	WT	WT	WT	WT	WT	√	
T20	77	M	Ⅳ	N/A	N/A	N/A	N/A	N/A	N/A	N/A	N/A	√	

Abbreviations: F, female; IDH, isocitrate dehydrogenase; LOH, loss of Heterozygosity; M, male; MGMT, O6‐methylguanine‐DNA methyltransferase; MT, mutant type; N/A, The patient was not tested for genes; TERT, telomerase reverse transcriptase; WT, wild type.

### 2.4 Isolation and culture of primary glioblastoma cells

Tumor specimens were subjected to primary cell isolation immediately after resection. The tissues were washed with PBS containing penicillin‐streptomycin to remove fat and connective tissue, cut into small pieces, and then incubated at 37°C for 0.5–1 h with DMEM medium containing appropriate amount of trypsin. After the digestion was terminated, the cell suspension was filtered and centrifuged. Cell pellet was resuspended and transferred to a 25 cm^2^ cell culture flask. The primary cells <4 passages were used for the experiment.

Primary cells were identified using GFAP immunofluorescence staining. Cells were fixed, blocked, permeabilized, and incubated with anti‐GFAP diluted with 1% BSA (abcam ab68428, 1:250) at 4°C overnight. Isotype IgG was used as a negative control. After wash, cells were stained with rabbit anti‐IgG H&L (Alexa Fluor 488^®^, abcam ab150077, 1:1000) at room temperature for 1 h. Cell nuclei were stained with Hoechst. Cells were mounted and then imaged using the PerkinElmer Vectra 3.

### 2.5 Transwell experiment

The treated and control cells were collected to prepare a single cell suspension and then centrifuged at 1,200 rpm for 5 min. The supernatant was discarded. The pellet was resuspended in 100 μL of serum‐free medium and mixed well. Cells were then added into the upper transwell. The lower chamber was filled with 600 μL of media containing 20% FBS. After 6 h of incubation, the media and cells in the upper chamber were wiped off. The upper chamber was fixed in 4% paraformaldehyde for 10 min and stained with Hoechst (Biyuntian C1025) for 15 min. Cells were imaged under a fluorescent microscope (Nikon Ts‐2). Cells in four fields were counted in each well, and the average values were calculated. The experiment was repeated three times independently.

### 2.6 Animals

Male Wistar rats (6–8‐week‐old, 150 ± 20 g) were purchased from Beijing Vital River Laboratory Animal Technology Co. Ltd. All rats were kept and fed in the laboratory animal center of the Institute of Biophysics, Chinese Academy of Sciences in Beijing. Cages, bedding, and food were sterilized and changed regularly. Protocols for animal handling, experimentations, and post‐surgery care were approved by the Institutional Animal Care and Use Committee of the PLA General Hospital and performed in strict accordance with the National Institute of Health ethical guidelines for animal care and use. All animal data reporting followed the ARRIVE 2.0 guidelines.^25^

Rats were shaved under isoflurane inhalation to ensure that the head top and jaw where the electrode attached were hairless before surgery. The electrodes were installed on the 3rd day after in situ inoculation of tumor cell suspension. The experiment was divided into three groups: the random sequence output group, fixed sequence output group, and control group. Blood and tissues were collected at specific times. A timeline of the experimental design is shown in Figure 5A. Rats that had severe neurological dysfunction were euthanized and excluded.

### 2.7 Establishment of glioma orthotopic transplantation model in rats

Animals were anesthetized with isoflurane inhalation and fixed on a stereotactic frame. After sterilization, an incision was made approximately 1 cm from the sagittal line (avoiding the attached electrodes). A small burr hole (diameter of 1 mm) was drilled using a dental drill on the right frontal bone. A 26‐G Hamilton syringe was used to inject a 5 μL C6 glioma cell suspension (containing 5 × 10^5^ cells) into the caudate nucleus according to the following coordinates: 1 mm anterior, 3 mm lateral to the bregma, and 6 mm below the skull (with a 1 mm withdrawal later). The cell suspension was slowly injected at a rate of 1 μL/min. The needle was maintained for 5 min after injection and retracted at 2 mm/min. The burr hole was sealed with sterilized medical bone wax, and the skin was sutured.

### 2.8 Electrode installation

Rats were anesthetized with isoflurane inhalation. The electrodes were fixed as shown (Figure S2Ca–d). As shown in Figure S2A,C, 1–2, 3–4, and 5–6 were pairs of positive and negative electrodes, generating 3 electric fields that were perpendicular to each other. For fixed sequence output, the electrodes were installed at locations 1–4. For random sequence output, the electrodes were installed at all 6 locations. The wires were fixed to the back of the rats. A transparent head cover was applied to each rat. The TEFT treatment (200 kHz) was applied continuously for more than 20 h per day. The electrodes were replaced every 2 days. The average temperature of the electrode was maintained at 36.2°C throughout the experiment. A sensor automatically sounded an alert when the temperature exceeded 40°C, and the equipment stopped working to ensure that the experiment was carried out safely and effectively.

Fixed sequence output: The voltage for 1–2 and 3–4 directions was 20Vpp. Frequency was 200 kHz, sinusoid, bi‐directional output. Switching time was 1 s. Random sequence output: The voltage for 1–2 and 3–4 directions was 20Vpp. Frequency was 200 kHz, sinusoid. The voltage for 5–6 directions was 27Vpp. Frequency was 200 kHz, sinusoid. There were three random direction outputs. Switching time was 1 s.

### 2.9 Measurement of electric field intensity

Rats were anesthetized with isoflurane inhalation. Two small burr holes, 0.73 cm apart, were drilled on the left and right parietal bones. Two insulated probes were inserted 0.8 cm below the skull. One end (1 mm long) of the probe was unwrapped and was used to detect electric signals. The other end of the probe was connected to an oscilloscope and used to detect the voltage and wave curve of the signal (Figure S2Ba–b). Similarly, probes were placed 0.5 cm below the temporal parietal bone as described above. The distance between two probes was 0.41 cm. One end of the probe was used to detect electric signals. The other end of the probe was connected to an oscilloscope and used to detect the voltage and wave curve of the signal (Figure S2Bc–d).

### 2.10 Magnetic resonance imaging (MRI) and tumor volume measurement

Rats were anesthetized and positioned on an animal cradle with a stereotaxic head holder. Gadolinium diamine (0.6 mmol/kg) was injected through the tail vein. T1‐weighted images of the tumor were acquired by a Ingenia 3.0T‐PHILIPS MRI (parameters in Table 2). The length and width of the tumor were measured using the Syngo fastview DICOM imaging device. Tumor volume was calculated with the following formula: Tumor Volume=length × width^2^/2 (mm^3^).^26^

**TABLE 2:** The parameter of magnetic resonance sequence

	TE (millisec)	TR (millisec)	Matrix	Field of view (mm)	Thickness (mm)	Number of excitations	Imaging time (min)
T1W	23	899	256 × 256	100 × 100	2.0	4	2:34
T2W	96	2,510	256 × 256	130 × 130	1.5	4	3:45

Abbreviations: T1W, T1‐weighted image; T2W, T2‐weighted image; TE, time of echo; TR, time of repetition.

### 2.11 Dermatological side effects

The dermatological side effects were evaluated according to the criteria below^27^ (Figure S3D): Animals without any dermatological side effects were scored as 0.

Grade I side effect was scored 1. The symptoms were shown as contact dermatitis, including local erythema, edema, and small hemorrhagic spots.

Grade II side effect was scored 2. The symptoms were shown as secondary local and invasive skin erosion with clear tissue exudate.

Grade III side effect was scored 3. The symptoms were shown as larger areas of skin ulceration. The base of the ulcer was clean, necrotic, granulated, or with small bleeding.

Grade IV side effect was scored 4. The symptoms were shown as Grade III skin side effect with concurrent skin or soft tissue infection. Yellow purulent discharge was observed around skin erosion.

### 2.12 Immunohistochemistry

4% PFA‐fixed tissues were paraffin embedded and sectioned into serial slices of 5 μm thickness. After permeabilization, antigen retrieval, and blocking, the brain slices were incubated with primary antibodies anti‐Ki‐67 (Abcam, ab 16667, 1:1000), anti‐CD8 (Abcam 1:1500), and cleaved caspase‐3 (CST Asp175 1:2500) diluted with 1% goat serum (Vector Laboratories) in PBS overnight. The slices were then incubated in the secondary antibody for 1 h and stained with the ABC HRP kit (Vectastain). After washing, the bound complex was visualized by incubating it with DAB (3,3‐diaminobenzidine). Images were obtained by Nikon‐Eclipse Ti. Image analysis was performed using Image J 1.51j8 with IHC Profiler to calculate the positive stained areas and to count the number of positive cells.

### 2.13 Hematoxylin and eosin (H&E) staining

The slides with cells were fixed with 4% paraformaldehyde for 20 min and washed with PBS. Cells were then stained with hematoxylin for 15 min, followed by eosin staining for 5 min. Cells were mounted on the slide using neutral balsam. Four fields in each slide were imaged using a microscope (Nikon‐Eclipse Ti) under a 20X lens. ImageJ V1.8.0 was used to count the cells and calculate the average cell surface area. The experiment was repeated 3 times independently.

### 2.14 Tumor electric field treatment for primary glioma cells

Primary cells in the logarithmic growth phase and within 3 passages were used for electric field treatment. The electric field frequency setting is displayed in Appendix 1. Cells were digested with 0.25% trypsin, centrifuged, and resuspended to prepare a single cell suspension. Cells were counted using a cell counter (Nexcelom Cellometer Mini). Cell diameter was measured.

### 2.15 Statistical analysis

GraphPad Prism 8.2.1 was used for statistical analyses. All data were expressed as mean ±standard deviation. The Student's *t* test was used to compare two groups for continuous variables with normal distribution. The Mann‐Whitney *U* rank sum test was used for continuous variables with non‐normal distribution. The differences in means across multiple groups were analyzed using two‐way analysis of variance (ANOVA) and Tukey *post hoc* analysis for data with normal distribution. Survival was estimated using the Kaplan‐Meier method, and the survival curve was compared using the log‐rank test. The difference was considered statistically significant when *p* < 0.05.

## 3 RESULT

### 3.1 TEFT inhibits the proliferation of glioblastoma cells in cultures

A tumor electric field treatment system was built for the current study. For *in vitro* experiments, two different sets of electric field outputs were applied to cell cultures, respectively, including the fixed sequence mode (Figure 1A) and the random sequence mode (Figure 1B). In the fixed sequence mode, the electric field is applied in two different directions, which alternate every second; in the random sequence mode, the electric field is applied in three different directions that are switched randomly every second.

#### 3.1.1 The anti‐tumor effect of TEFT with different treatment duration

**FIGURE 2:**
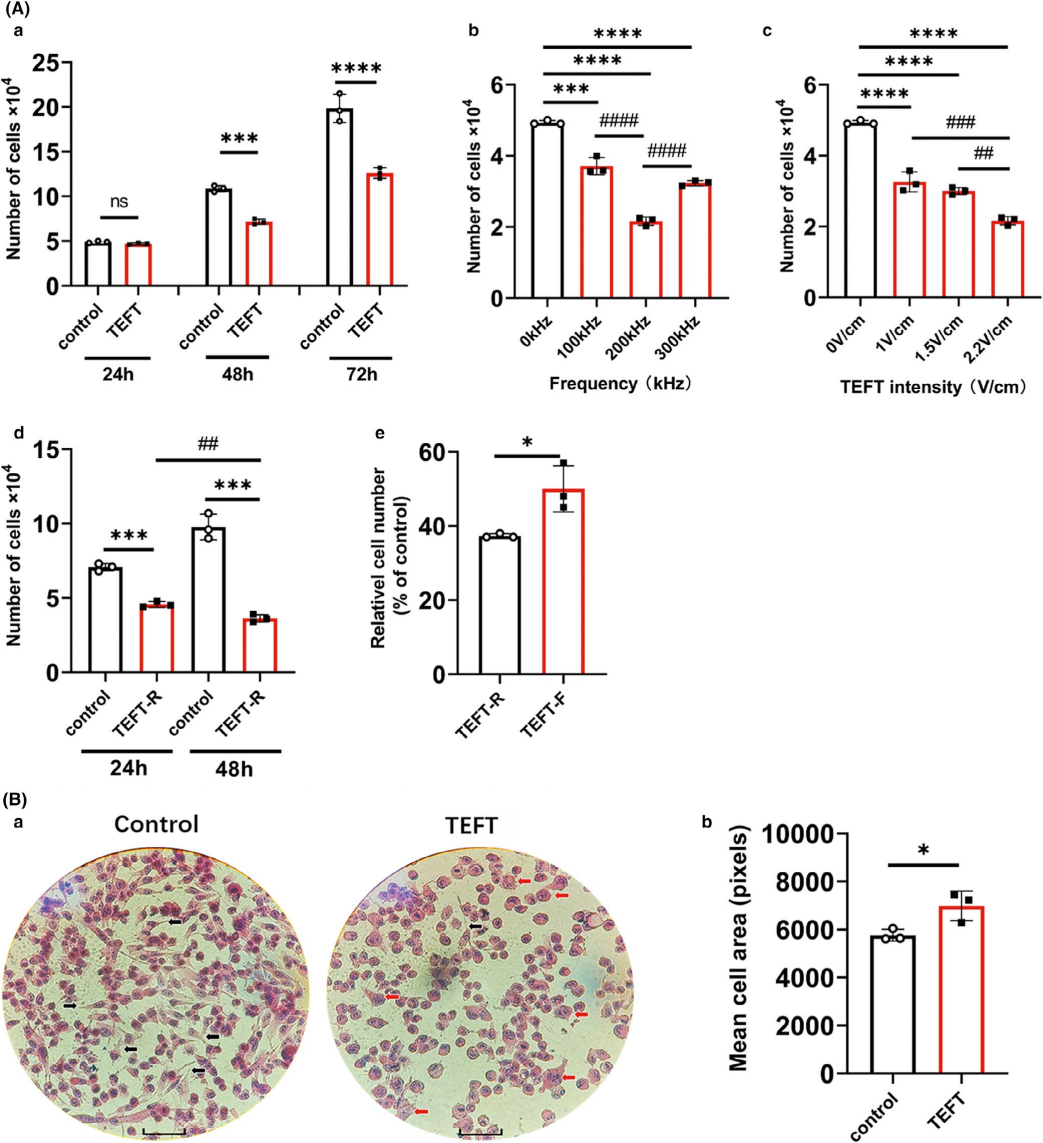
Tumor‐suppressing effect *in vitro* under different parameters and output modes of tumor electric field therapy system. (Aa) Glioblastoma cells were treated with TEFT for 24 h, 48 h, or 72 h, using a fixed sequence of electric field treatment with a frequency of 200 kHz and a field strength of 2.2V/cm. (Ab) Glioblastoma cells were treated with TEFT for 48 h with 2.2 V/cm field strength and frequency of 100, 200, or 300 kHz. (Ac) Glioblastoma cells were treated with TEFT for 48 h with 200 kHz frequency and field strength of 1, 1.5, or 2.2 V/cm. (Ad) Glioblastoma cells were treated with TEFT for 24 h and 48 h, respectively, with fixed field strength 2.2V/cm and frequency of 200 kHz but a randomly sequential output mode. (Ae) Glioblastoma cells were treated with TEFT for 48 h with fixed field strength 2.2V/cm and frequency of 200 kHz, comparing the effect of fixed vs. randomly sequential electric field. (Ba) Representative microscopic field of HE‐staining of glioblastoma cells that were treated with TEFT or control condition for 48 h with fixed field strength 2.2V/cm and frequency of 200 kHz and a fixed sequential electric field. Black arrows showed tentacles, the red arrows showed apoptosis. Scale bar =50 μm. (Bb) Quantification of glioblastoma cells that were treated with TEFT or control condition for 48 h with fixed field strength 2.2V/cm and frequency of 200 kHz and a fixed sequential electric field. All experiments were performed three times using independent preparations, and the number of glioblastoma cells under each experimental condition was quantified by cell counting. All data are mean ±standard deviation, * or #*p* < 0.05, ** or ##*p* < 0.01, *** or ###*p* < 0.001, **** or ####*p* < 0.0001, * represents the statistical comparison between experimental group and its control group, # represents the statistical comparison between experimental groups. ANOVA and *Tukey post hoc* analysis (Aa–d); Student's *t* test, unpaired (Ae, Bb)

The U251 cell line of glioblastoma cells was treated with TEFT for 24, 48, or 72 h, using a fixed sequence electric field output at frequency of 200 kHz and field strength of 2.2V/cm. Cells were counted after treatment, and cell morphology was observed after H&E staining. The number of cells in the control group doubled every 24 h, while the cell proliferation in the TEFT‐treated groups was substantially slower. Twenty‐four hours of treatment had no significant effect on cell proliferation (*p* = 0.1557, *n* = 3), whereas there were significant differences between control and TEFT‐treated groups at both 48 h (*p* = 0.0003, *n* = 3) and 72 h (*p* < 0.0001, *n* = 3) time points (Figure 2Aa). However, no statistical differences in tumor suppression were found between the 48 h and 72 h groups.

#### 3.1.2 Tumor suppression effects of different electric field frequencies

Tumor electric field treatment of frequencies at 100, 200, and 300 kHz with a fixed field strength of 2.2V/cm was tested. All three frequencies significantly inhibited tumor cell proliferation (100 kHz vs. 0 kHz, *p* = 0.0011; 200 kHz vs. 0 kHz, *p* < 0.0001; 300 kHz vs. 0 kHz, *p* < 0.0001, data from 3 independent experiments), and the frequency at 200 kHz appeared to generate the maximal cell‐killing effects (Figure 2Ab).

#### 3.1.3 The tumor suppression effects of TEFT with different field strengths

To determine the optimal field strength, we applied electric fields with three different field strengths (1.0, 1.5, and 2.2V/cm) with a fixed frequency at 200 kHz. TEFT for 48 h at all three strengths significantly inhibited tumor cell proliferation (1.0V/cm vs. 0V/cm, *p* = 0.0003; 1.5V/cm vs. 0V/cm, *p* < 0.0001; 2.2V/cm vs. 0V/cm, *p* < 0.0001, data from 3 independent experiments). The anti‐cell proliferation effects of TEFT at 2.2V/cm were greater than 1.0 or 1.5V/cm (Figure 2Ac), exhibiting a field strength‐dependent efficacy.

#### 3.1.4 The anti‐tumor effect of different TEFT output modes

A previous study suggested that increasing the alternating electric field from one direction to three directions could improve the anti‐proliferation effect of TTFields.^1^ Therefore, we tested the effect of three‐directional electric fields by randomly switching every second (TEFT‐R) against U251 cells. TEFT‐R at the optimal frequency (200 kHz) and strength (2.2V/cm) significantly inhibited cell proliferation at 24 h (*p* = 0.0002) and 48 h (*p* = 0.0003) after treatment (Figure 2Ad).

We then compared the anti‐proliferation effect between TEFT‐R and the bi‐directional electric fields that underwent fixed alternations every second (TEFT‐F). The results revealed that, after 48 h of treatment, TEFT‐R exhibited significantly greater anti‐proliferation effect than TEFT‐F (*p* = 0.0249, Figure 2Ae).

#### 3.1.5 TEFT‐treated cells exhibit morphological changes

U251 cells after treatment with TEFT for 48 h exhibited morphological changes that were characterized by the loss of tentacles and enlargement of cell bodies (Figure 2Ba). Quantitative analysis revealed that the surface area of U251 cells was significantly increased compared with control cells (*p* = 0.0335, Figure 2Bb).

### 3.2 TEFT suppresses the proliferation of patient‐derived primary glioblastoma cells *in vitro*

In the next set of experiments, we determined whether TEFT could suppress cell proliferation of primary glioblastoma cells. To this end, we collected resected specimens from a total of 20 patients who underwent surgical treatment of glioma in the Department of Neurosurgery at the PLA General Hospital (Table 1). All of these patients were diagnosed as glioblastoma (WHO stages III‐IV) and pathologically confirmed after surgery. Both new (18 cases) and relapsed (2 cases) patients were included. Patients were presented with different genetic traits, including MGMT methylation (7 cases), LOH deletion at 1p (1 case) or 19q (3 cases), IDH mutations (1 case), TERT mutations (7 cases), and BRAF mutation (1 case).

**FIGURE 3:**
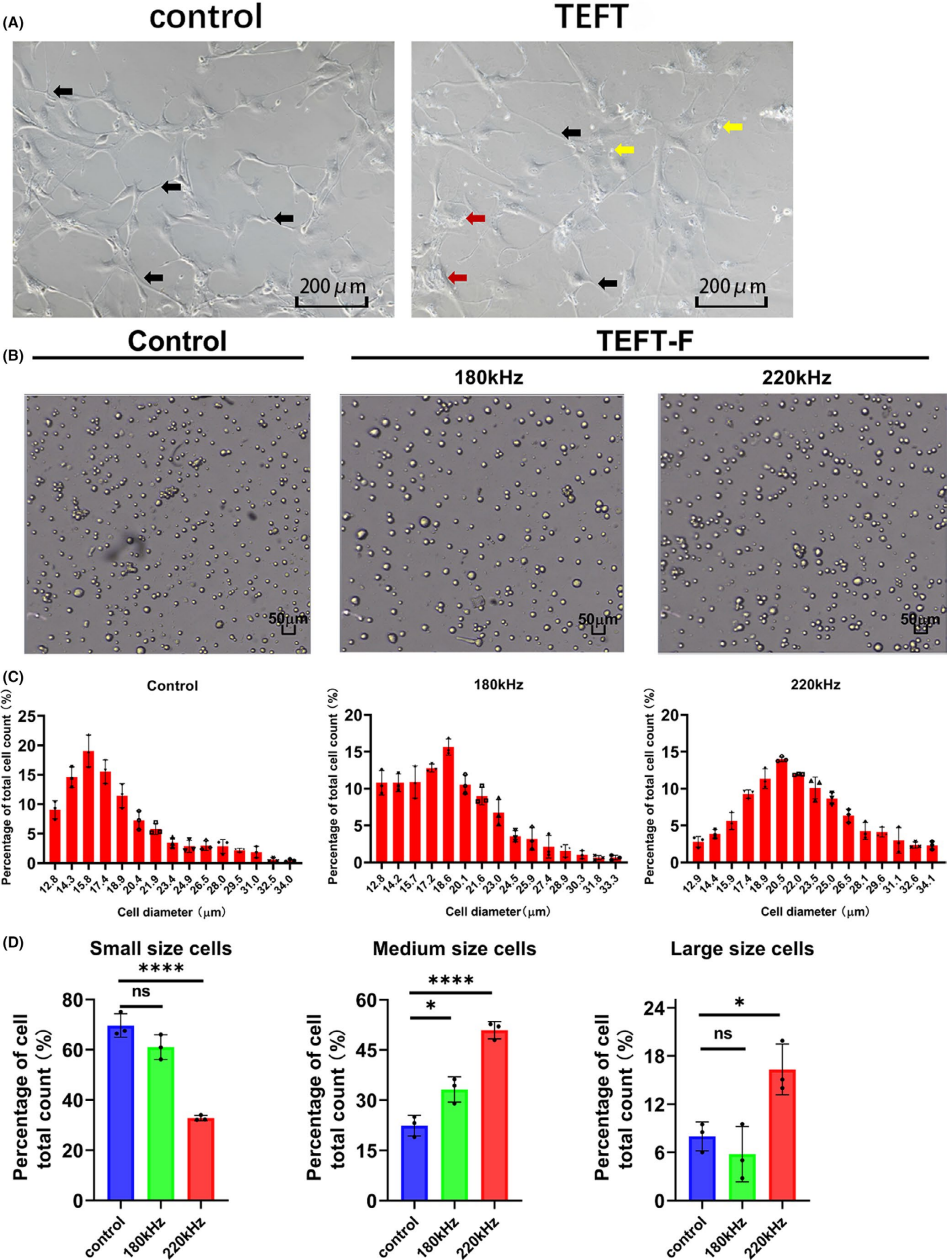
Cell size changes of primary glioblastoma cells after TEFT treatment. Primary glioblastoma cells were treated with TEFT for 72 h using a fixed sequence of electric field with a frequency from 100 to 220kHz and a field strength of 2.2V/cm. (A) Representative phase‐contrast microscopic image showing cellular enlargement in cultures after TEFT, black arrows showed tentacles, the red arrows showed apoptosis, and the yellow arrows showed cell bubbles. (B) Representative phase‐contrast microscopic images of cell suspensions after TEFT at 220 kHz and 180 kHz, respectively, or control treatment. Scale bar =50 μm. (C) Individual cell sizes were measured using a cell counter in cell suspensions after TEFT at 220 kHz and 180 kHz, respectively, or control treatment. All cells are divided into 15 groups based on the different diameters as indicated, and data expressed as percentage of total cell counts at each of the diameters. (D) Statistical analysis of cell sizes under the three experimental conditions; cell sizes are grouped in three size ranges, including small size (diameter of 12.9–18.9 μm), medium size (diameter of 20.5–26.5 μm), and large size (diameter of 28.1–34.1 μm). *p < 0.05, **p < 0.01, ***p < 0.001, ****p < 0.0001. *Tukey post hoc* analysis (D)

Primary cells were prepared from fresh specimens and cultured until they entered the logarithmic growth phase. The cells were treated with TEFT at frequencies of 100, 150, 180, 200, or 220 kHz and electric field strength of 2.2V/cm for 72 h. Microscopic examination revealed that TEFT‐treated primary glioblastoma cells blistered and grew sparsely (Figure 3A). TEFT‐treated cells (frequency 180 kHz or 220 kHz) appeared to be enlarged compared with the non‐treated cells (Figure 3B). Quantitative analysis confirmed that TEFT‐treated cells showed overall increases in cell diameters. Figure 3C illustrates the relative cell distribution (% of total cell counts) at each of the cell diameter scales after control treatment or TEFT at 180 kHz or 220 kHz. Figure 3D shows the relative cell numbers (% of total cell counts) with small diameters (12.9–18.9 μm), medium diameter (20.5–26.5 μm), and large diameter (28.1–34.1 μm) after control treatment or TEFT at 180 kHz or 220 kHz.

#### 3.2.1 TEFT effectively inhibits the proliferation of primary glioblastoma cells at different optimal frequencies

**FIGURE 4:**
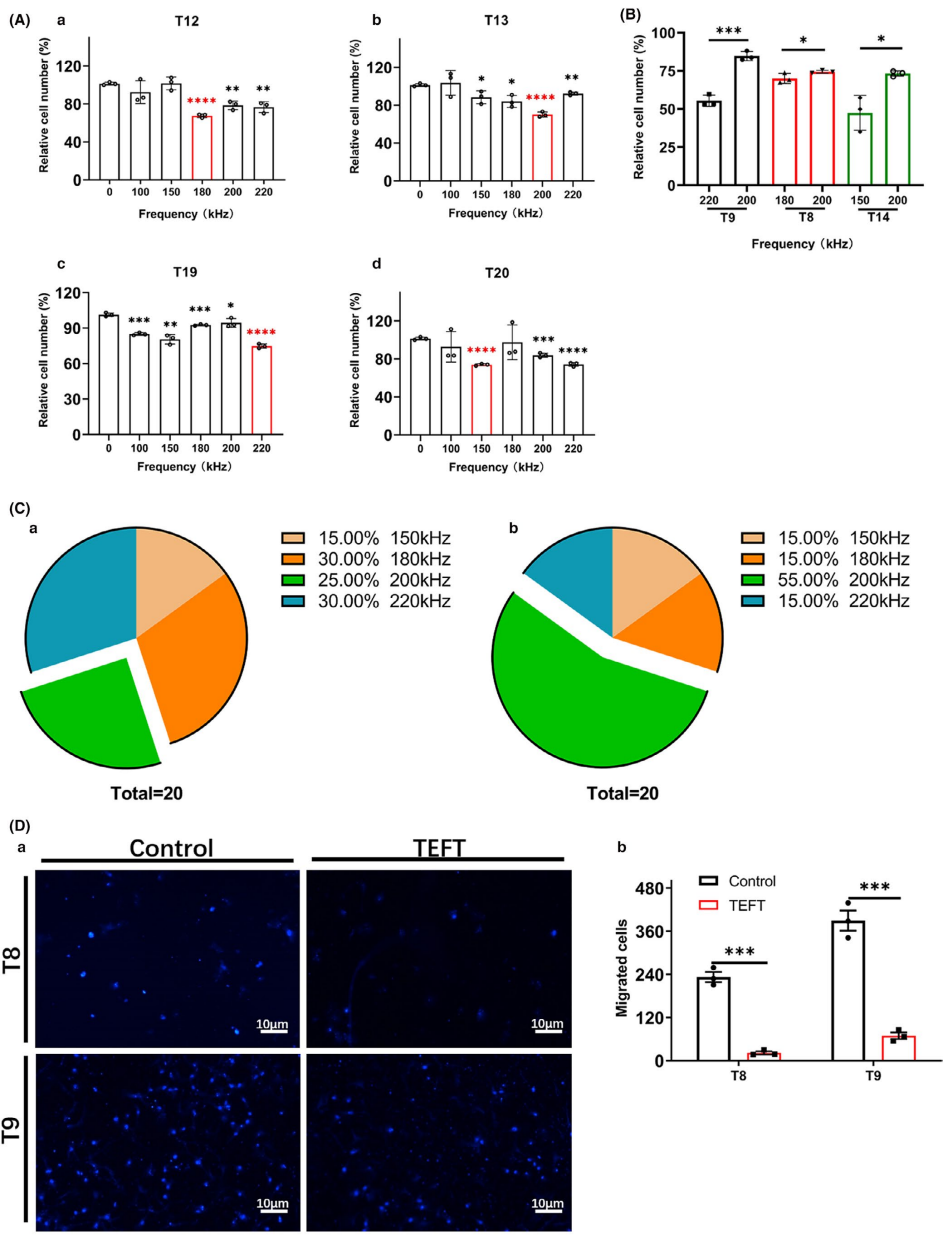
Frequency‐ and field strength‐dependent inhibition of glioblastoma cell proliferation and invasion by TEFT‐F. Cultured primary cells were treated with TEFT for 72 h using a fixed sequence of electric field with a frequency of 200 kHz and a field strength of 2.2V/cm. (A) Different frequencies of TEFT resulted in different levels of inhibition on cell proliferation in different primary glioblastoma cells (T12, T13, T19, and T20). The optimal frequencies are 180 kHz for T12 cells (Aa), 200 kHz for T13 cells (Ab), 220 kHz for T19 cells (Ac), and 150 kHz for T20 cells (Ad), which are shown in red columns. (B) The bar graph illustrated the optimal frequencies of TEFT for T9 (220 kHz), T8 (180 kHz), and T14 (150 kHz) cells and their comparison to the anti‐proliferation effect at 200 kHz. (C) The pie chart in the left panel shows the percentages of primary cells from the total 20 cases of glioblastoma patients at their respective optimal frequencies (150, 180, 200, and 220 kHz) of TEFT(Ca). The pie chart in the right panel shows that comparing the optimal frequency of non‐200 kHz cells with their optimal frequency, those with no statistical difference were included in the 200 kHz group, and those with differences were included in the original group, and the proportion of cell lines with the optimal frequency was redrawn (Cb). (D) Results of transwell experiments, in which primary glioblastoma cells that migrated through the transwells were stained with Hoechst (Da) and quantified (Db). All data are mean±SD, from three independent experiments, *p < 0.05, **p < 0.01, ***p < 0.001, ****p < 0.0001. ANOVA and *Tukey post hoc* analysis (A); Student's *t* test, unpaired (B, Db)

Primary glioblastoma cells from different patients showed different sensitivity to electric field frequencies (Figure 4A). Figure S1 shows the results of all 20 primary cell preparations in responses to TEFT at a range of frequencies from 100 kHz to 220 kHz. The optimal frequency (to achieve the maximal proliferation‐suppression effect) was different among all primary cell preparations: 150 kHz for 3 cases (15% of all cases), 180 kHz for 6 cases (30% of all cases), 200 kHz for 5 cases (25% of all cases), and 220 kHz for 6 cases (30% of all cases). In 2 cases (T6 and T10), TEFT at frequency of 200 kHz failed to suppress cell proliferation (Figure S1). For the 15 cases in which the optimal frequency was not 200 kHz, we compared the effect at their respective optimal frequency with that of 200 kHz (Figure 4B) and those that showed no statistical difference were reclassified into the 200 kHz group. The proportion of the 200 kHz group increased to 55% after this reclassification (Figure 4C).

#### 3.2.2 TEFT effectively inhibits the proliferation of primary glioblastoma cells regardless of different genetic traits

Eleven of the 20 specimens collected in this study underwent 6 tumor genetic tests (Table 1). Taking all experimental results together, we found that primary glioblastoma cells with MGMT methylation, and IDH, TERT, or BRAF mutations, or 1p/19q co‐deletions were all sensitive to TEFT and showed no resistance compared to cells without any of the above 6 genetic traits. Each of the primary cell preparations with a specific genetic trait remains sensitive to one or more frequencies within the range of 100–220 kHz (Figure S1).

#### 3.2.3 TEFT inhibits the invasiveness of primary glioblastoma cells

We further assessed the invasive phenotype of primary glioblastoma cells after TEFT using the transwell setting. Two representative primary cell preparations (T8 and T9) were treated with their optimal frequencies (180 and 220 kHz, respectively) of electric fields for 72 h. The results revealed that TEFT significantly suppressed the invasiveness of both primary cell preparations compared with their control groups (T8 vs. control, *p* = 0.0001; T9 vs. control, *p* = 0.0004, Figure 4D).

### 3.3 TEFT suppresses tumor growth in an *in vivo* rat model of glioblastoma implantation

To determine the *in vivo* effect of TEFT, we established a rat model of glioblastoma by inoculating C6 glioma cells (cell number, 5 × 10^5^) into the caudate nucleus. The TEFT was set up for treatment in rats as illustrated (Figure S2), which allows either the TEFT‐F or TEFT‐R mode.

#### 3.3.1 Assessment for the field strength of TEFT in rat brain

The effective field strength for TEFT was determined through a probe inserted into the brain. For the TEFT‐F mode (1–2 and 3–4 direction, Figure S2), the distance between two electrodes was 0.73 cm and the voltage was 1.816 Vpp. According to the formula Em =U/d, the electric field strength was 2.49Vpp/cm. The effective field strength was 0.88V/cm, per the formula E=Em/√2. For the TEFT‐R (Figure S2), the distance between two probes was 0.41 cm and the voltage was 1.023 Vpp. The electric field strength was 2.50Vpp/cm, and the effective field strength was 0.88V/cm.

#### 3.3.2 The impact of TEFT‐R on general condition of rats

**FIGURE 5:**
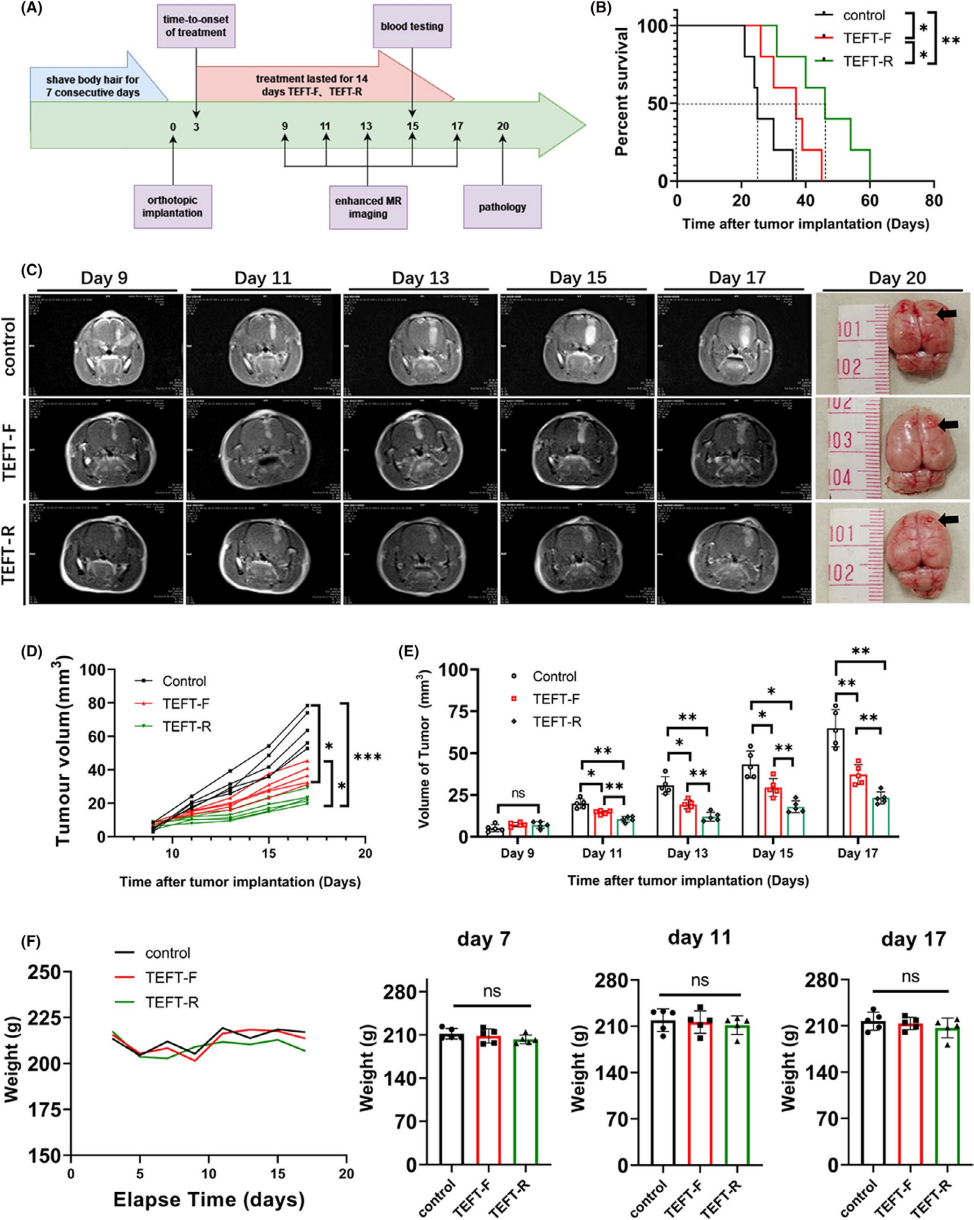
The random sequential output mode inhibits tumor growth and prolongs the survival time of tumor‐bearing rats. (A) Random sequential output mode *in vivo* experiment flow chart. (B) The survival curves of tumor‐bearing rats that received TEFT‐F, TEFT‐R or control treatment. (C) Representative images of enhanced magnetic resonance scanning on rats at 9, 11, 13, 15, and 17 days after tumor inoculation and received TEFT‐F, TEFT‐R or control treatment. The last column of photographs shows brain gross view at 20 days after tumor loculation. The arrows point to the tumor site. (D,E) Quantification of tumor volume based on MRI at 9, 11, 13, 15, and 17 days after tumor inoculation in rats that received TEFT‐F, TEFT‐R, or control treatment. (F) Body weight changes in tumor‐bearing rats at 3–17 days after tumor inoculation. All data are expressed as mean±standard deviation, *n* = 5/group, *p < 0.05, **p < 0.01, ***p < 0.001, ****p < 0.0001. Two‐way ANOVA (D and E) or the Kaplan‐Meier test (B)

Rats with glioma orthotopic transplantation showed signs of reduced intake of food and drink, reduced body weight, and less activity, all of which improved at day 3 after transplantation. Rats were then randomly assigned to TEFT‐F, TEFT‐R, and control groups. With the growth of a tumor and installation of electrodes, all 3 groups of rats exhibited signs of reduced intake of food and drink, reduced body weight, yellowish hair without gloss, hunched, reduced activity, or tilting to one side when moving. The body weight loss peaked at 5 days after transplantation, and then gradually recovered. At 7, 11, and 17 days after transplantation, three experimental groups showed differences in body weight (control>TEFT‐F>TEFT‐R), but the differences did not reach statistical significance (*p* > 0.05, Figure 5F).

No limb convulsions or epileptic seizures were observed during TEFT treatment. Random measurements during treatment showed that the temperature of electrodes fluctuated between 36.2°C and 39.6°C.

#### 3.3.3 TEFT‐R effectively inhibits tumor growth in tumor‐bearing rats

The timelines for the TEFT efficacy studies are illustrated (Figure 5A). TEFT significantly prolonged the overall survival time compared with control rats (Figure 5B): TEFT‐F vs. control group, *p* = 0.0415 and TEFT‐R vs. control group, *p* = 0.0064. TEFT‐R appeared to be more effective than TEFT‐F in prolonging the survival time of tumor‐bearing rats (*p* = 0.0471, Figure 5B).

The effect of TEFT on tumor growth was assessed by measuring tumor volume by MRI. MRI was performed at 9, 11, 13, 15, and 17 days after C6 glioma inoculation (Figure 5C). There was no significant difference in tumor volume among the three experimental groups at 9 days after glioma inoculation (*p* > 0.05). Both TEFT‐F and TEFT‐R markedly reduced tumor volume at 11, 13, 15, and 17 days after glioma inoculation (Figure 5D,E). Notably, TEFT‐R showed greater effects in reducing tumor volume than TEFT‐F (Figure 5D,E).

#### 3.3.4 TEFT‐R promotes apoptosis and CD8+ T‐cell infiltration *in vivo*

**FIGURE 6:**
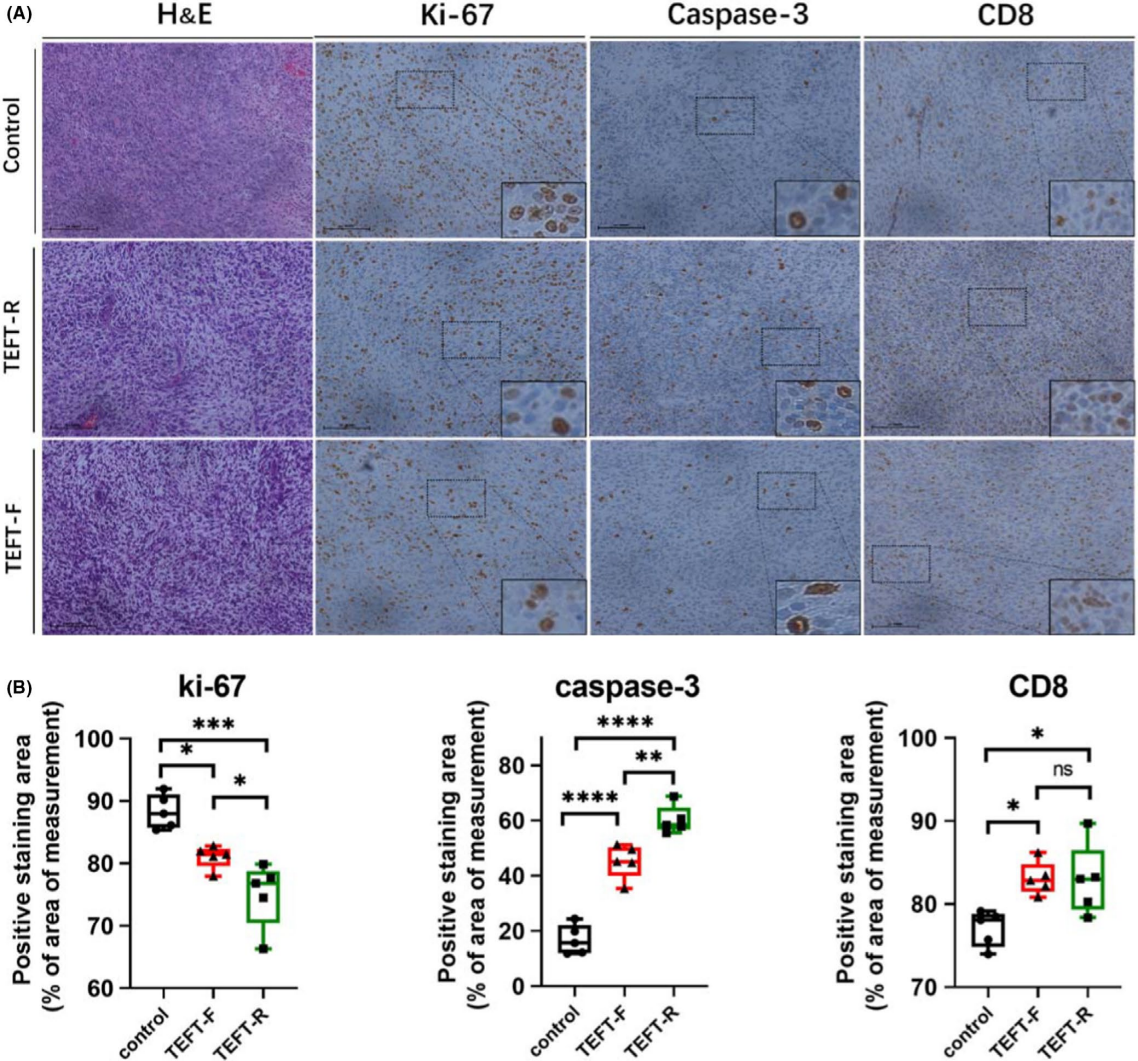
Effects of TEFT on markers for cell proliferation (Ki‐67), apoptosis (active caspase‐3), and T effector cell infiltration (CD8) in inoculated glioblastoma in rats. Rats were inoculated with tumor cells, and then subjected to TEFT‐F, TEFT‐R, or control treatment. Rats were sacrificed at 20 days after tumor inoculation. (A) Representative microscopic images of immunohistochemical staining for Ki‐67, Caspase‐3, and CD8 in cross‐tumor sections. The inserts are high‐power view of positive cells in the selective areas indicated by the rectangle boxes. Scale bar =100 μm (B) Quantitative data on immunoreactive cells for Ki‐67, caspase‐3, and CD8. All data are expressed as mean±SD, *n* = 5 per group. *p < 0.05, **p < 0.01, ***p < 0.001, ****p < 0.0001. One‐way ANOVA and *Tukey post hoc* analysis

It has been reported previously that TTFields inhibit tumor cell proliferation,^28^, ^29^, ^30^ promote apoptosis,^31^, ^32^, ^33^ and increase CD8^+^ T‐cell infiltration in the tumor.^14^ To determine whether TEFT suppresses glioblastoma growth by similar mechanisms, we performed immunohistochemical staining on tumor specimens (Figure 6A). The results revealed that both TEFT‐R (vs. control group, *p* = 0.0002) and TEFT‐F (vs. control group, *p* = 0.0138) inhibited tumor proliferation as indicated by decreased numbers of KI67^+^ cell in TEFT‐treated animals (Figure 6A–B). TEFT also promoted apoptosis and the infiltration of CD8^+^ T cells into tumor, demonstrated by increased expression of the active form of caspase‐3 and a specific marker of CD8^+^ T cells in the tumor compared with the control rats (Figure 6A–B). In comparison, TEFT‐R showed greater effects in inhibiting tumor proliferation (vs. TEFT‐F, *p* = 0.0383) and promoting cell apoptosis (vs. TEFT‐F, *p* = 0.0019).

#### 3.3.5 Side effects of TEFT

The impacts of TEFT on liver and kidney functions were evaluated. TEFT treatment showed no effect on serum glutamic oxaloacetic transaminase (AST), alanine aminotransferase (ALT), serum creatinine, white blood cell count, or platelet count (*p* > 0.05, Figure S2A). TEFT‐R increased serum creatinine (vs. control group, *p* = 0.0087), red blood cell count (vs. control group, *p* = 0.0151), and hemoglobin levels (vs. control group, *p* = 0.0302, Figure S2A). TEFT‐F increased serum creatinine level (vs. control group, *p* = 0.0368, Figure S2A). Pathological examinations revealed no gross changes in vital organs of TEFT‐treated rats, including the liver, kidney, and brain (Figure S2B).

Skin reaction is a common adverse reaction observed after TEFT. We observed local contact dermatitis and skin erosion in animals treated with TEFT‐F (occurring in 40% rats) or TEFT‐R (occurring in 80% rats) at 11 days after glioma inoculation (Figure S2E). Grade III skin reactions were observed in 10% of TEFT‐R treated rats and in 20% of TEFT‐F‐treated rats at 13 days after glioma inoculation (Figure S2E). Skin side effects peaked at 15 days after glioma inoculation, with 100% of TEFT‐R‐treated rats showing skin reactions (40% of which having grade I reactions, and 60% having grade II reactions), with 80% of TEFT‐F‐treated rats showing skin reactions (62.5% of which having grade I reactions and 37.5% of which having grade II reactions) (Figure S2E). Skin reactions gradually subsided at 17 days after daily topical treatment using local disinfectant and corticosteroids (Figure S2C,E), leaving 40% of TEFT‐R‐treated rats and 20% of TEFT‐F‐treated rats with grade I skin reactions (Figure S2E).

## 4 DISCUSSION

The Inovitro™ system (Novocure)^24^ is widely used for *in vitro* tumor electric field therapy. In this study, we tested the TEFT and evaluated the effectiveness of this domestic instrument. Different times, frequencies, and field strengths of TEFT were tested. The results showed that domestic instruments can effectively inhibit the proliferation of glioma cell lines and achieve similar tumor suppression effects compared with the Inovitro™ system.

According to early research about TTFields, increasing the electric field direction from 1 to 3 can significantly improve the anti‐proliferation effect of TTFields.^1^ Therefore, we propose a treatment mode of random sequence electric field output (TEFT‐R). This mode provides three vector fields (with an angle of 60° for *in vitro* experiments and an angle of 90° for *in vivo* experiments). The TEFT‐R was adopted to cover more widely the random directions of mitotic axes of tumor cells in the treatment field. The random sequence output mode exhibits a significant tumor inhibitory effect within the first 24 h of treatment, which is faster than the fixed sequence mode. The 48 h treatment is more effective than the 24 h treatment. These advantages of the random sequence output mode are proved *in vitro* and *in vivo*.

Glioma cell line U251 is an immortal cell line that is commonly used for glioma research. It has the advantages of accessibility, rapid expansion, and low cultivation difficulty. However, immortal tumor cell lines have accumulated a large number of gene mutations in order to adapt to the *in vitro* environment,^34^ which makes it impossible to fully reflect the status of tumor cells *in vivo*. Secondly, cell lines are derived from a single clone and are unable to represent the heterogeneity of tumor cells. Therefore, we used primary cells derived from different glioma patients to evaluate personalized TEFT treatment.

We note that primary tumor cells treated with the sensitive frequencies of electric fields exhibit reduced protrusions and enlarged cell bodies. These changes were prominent in cells treated with the optimal frequency of TEFT. Similar phenomena were observed in the TEFT‐treated glioma cell line. Compatibly, R Schneiderman^35^ and Turner et al.^36^ also observed giant tumor cells in the tumor tissues collected from glioma patients whose tumor growth was effectively inhibited after combined electric field and temozolomide treatment. They believe that volume increase is a method for tumor cells to resist electric field treatment. By maintaining a larger volume, tumor cells become resistant to a fixed frequency of electric field. This resistance can be overcome by resetting the frequency and field strength, which have been confirmed in the studies of ovarian cancer cells.^37^

Taken together, we believe that the electric field interferes with mitosis^32^ in the early stage of treatment, leading to the electrophoresis of charged substances toward the cleavage furrow,^15^, ^38^ increased cell membrane pressure, and eventually cell blebbing or even rupture. Therefore, cell enlargement is more prominent in response to optimal frequency treatment. As the treatment progresses, continuous exposure to the electric field leads to changes in the genetic characteristics of tumor cells,^39^ and the tumor cells become resistant to electric field treatment by maintaining a larger volume. According to the theory that the frequency of TTFields is inversely proportional to the cell volume,^1^ the frequency should be adjusted during the electric field treatment, depending on the conditions of the patients, to further improve its therapeutic effects.

We find that primary cells with different genetic traits, including MGMT methylation, IDH, TERT, BRAF mutations, and 1p/19q co‐deletions, are all sensitive to TEFT. Combined with the results of genetic testing, primary glioma cells with MGMT non‐methylation (which predicts insensitivity to chemotherapy^40^, ^41^), 1p/19q heterozygous non‐co‐deletion, and IDH‐1/2 wild type (which indicate a poor clinical prognosis^42^, ^43^) are all sensitive to TEFT. However, additional primary glioblastoma cells with other molecular traits should be tested for efficacy of TEFT. More recent studies have identified several molecular alterations in glioblastoma that are associated with radio‐ or chemo‐resistance and poor prognosis in clinic, for example, B3GNT5,^44^ elevation of CXCL1^45^ or TRIB2 and MAP3K1,^46^ and COPB2.^47^ A demonstrated effectiveness of TEFT in primary cells with the above additional biomarkers would enhance the translational probability of this technology in clinical treatment of glioblastoma.

Our research shows that there are multiple sensitive frequencies for each cell line, and there are multiple cell lines that are sensitive to the same frequency. In addition, each cell line has a specific optimal frequency among all sensitive frequencies. In theory, this optimal frequency is the ideal treatment frequency for this patient. Interestingly, the optimal frequencies are not the same among primary cells collected from different patients. The primary cells with the optimal frequency of 200 kHz (F200 group) accounted for only 25% of total cell lines. Even after adding the cell lines whose optimal frequency is not 200 kHz but shows no significant difference in their response to 200 kHz, the F200 group still accounted for only 55% of the total cell lines. This suggests individual differences in the optimal frequency of TEFT. Although 200 kHz represents a common sensitivity, it is not the optimal frequency for all patients, and a few patients not even respond to it (2 cases are not sensitive to 200 kHz). This can explain the case resistance to TTFields in the clinic.^48^ A fixed frequency at 200 kHz will effectively inhibit tumor growth in 55% of patients; however, there are still many patients who may not even respond to this fixed frequency. Therefore, electric field therapy should be personalized so that nobody would miss their optimal therapeutic frequency.

We only collected 20 tumor specimens for a small sample *in vitro* study, of which only 11 cases were subjected to genetic detection. Our conclusions that primary cells of different genetic traits are all responsive to electric field treatment and that different cells have different optimal frequencies are based on this small sample study. However, the gene mutations in the development of glioblastoma are much more complicated and could not be fully represented by the data presented in this study. Second, the conclusions from our *in vitro* experiments need to be further verified by clinical trials. More patient clinical data and gene sequencing data should be collected and analyzed together with the results from clinical trials to further evaluate the overall efficacy of electric field therapy on glioma patients and screen molecular biomarkers for TTFields treatment.

Our in *vivo* experiments show that TEFT‐R has better tumor‐suppressive effect than TEFT‐F. However, is TEFT‐R better than TEFT‐F in all aspects? We find that the body weight of TEFT‐R‐treated rats is lower than the control at multiple time points, although the difference is not statistically significant. Additionally, the skin side effects are more severe in the TEFT‐R group than TEFT‐F group, which is manifested as more rats having grade II or higher skin reactions. Finally, blood tests reveal that TEFT‐R increases serum creatinine, red blood cell count, and hemoglobin levels. The serum urea level in all groups is within normal range but at its upper limit, probably due to reduced water intake. Therefore, TEFT‐R has more adverse impacts in rats, including body weight loss, skin reaction, and abnormal blood tests. Parameters of TEFT‐R need to be further optimized to ensure long‐term usage and better therapeutic effects.

There are several limitations in this study, especially the *in vivo* animal work. First, the current rat model of glioblastoma was produced by inoculation of the C6 cell line, which does not reflect the intra‐ and inter‐tumoral heterogeneity on molecular characteristics and sensitivities to therapies.^49^ While it has been a considerable challenge in the field to inoculate primary glioblastoma cells into rodent brain, we will need to establish such models in the future to identify the optimal treatment paradigms for TEFT. Second, the side effects of TEFT were only partially studied in the current study, with a focus on local skin reactions. However, other side effects could occur, for example, would TEFT aggravate peritumoral cerebral edema? The latter is a major contributor to neurological impairment and mortality in clinical glioblastoma.^50^ Future studies should be performed to examine the impact of different TEFT paradigms on brain edema that are associated with inoculated glioblastoma. Third, biomarkers for TEFT are not explored in this study; however, non‐invasive biomarkers are essential for testing a novel therapy in clinical trials. Several non‐invasive brain imaging technologies have been merging for such purposes, including novel MRI approaches that measure neurometabolic alterations^51^ or visualize the tumor microenvironment with specific parameters on oxygen metabolism and neovascularization.^52^, ^53^

## 5 CONCLUSIONS

The fixed sequence output TEFT effectively inhibits the proliferation and invasiveness of glioma cells with common genetic mutations. The TEFT random sequence output mode further improves the therapeutic effect on glioblastoma. Importantly, different primary glioblastoma cells are sensitive to specific frequencies of electric field.

The precision treatments are strongly advocated recently. Optimal treatment regimen should be designed according to the specific conditions of patients to achieve the best curative effect and minimize adverse reactions. For electric field therapy, optimizing treatment parameters according to the conditions of individual patients and improving the potability of the equipment are critical to further improve its overall efficacy.

## CONFLICTS OF INTEREST

The authors declare no conflicts of interest.

## AUTHOR CONTRIBUTION

D.Z., D.C., and J.L. isolated tumor tissues and cultured primary tumor cells, J.C., A.W., N.R., and S.L. were responsible for debugging the instruments and arranging the time for animal experiments. H.W., J.C., C.L., and Z.W. assist in MRI scanning. J.C., H.W., L.Y., and H.Y. establish tumor animal model, and X.Z., G.S., and J.L. organized ethical approval. A.W., S.W., L.T., F.R., Y.Z., D.S., Y.L., M.J., H.L., and J.L. helped to collect clinical samples and data. L.C., J.L., and Z.L. participated in data analysis and project design. H.W., L.Y., and H.L. wrote the manuscript. All the authors reviewed and accepted the contents of the article. H.W., L.Y., and H.L. equally contributed to this manuscript. L.C., J.L., and Z.L. share senior authorship.

## Supporting information

Fig S1

Fig S2

Fig S3

## Data Availability

All data associated with this study are present in the paper or the Supplementary Materials.

## APPENDIX 1

### Experimental methods

1.Parameter setting of immortal cell line experiment
Different duration in fixed sequence output: fixed frequency 200 kHz, field strength 2.2V/cm, exposed for 24, 48, and 72 h, respectively;
Different frequencies in a fixed sequence output: fixed time 48 h, field strength 2.2V/cm, processing frequency 100, 200, 200, and 300 kHz, respectively;
Different field strength in fixed sequence output: fixed time 48 h, frequency 200 kHz, treatment field strength 1, 1.5, and 2.2V/cm, respectively;
Random sequence output: fixed frequency 200 kHz, field strength 2.2V/cm, exposed for 24 h and 48 h.
2.Parameter setting of primary cell experiment
Different frequencies in fixed sequence output: fixed time 72 h, field strength 2.2V/cm, processing frequency 100,150,180,200, and 220 kHz, respectively.
