# Supplementary figures and images for: Exploring the efficacy of tumor electric field therapy against glioblastoma: An in vivo and in vitro study
Source: CNS Neurosci Ther. 2021 Oct 28;27(12):1587–604. doi: 10.1111/cns.13750 (PMC8611775; doi:10.1111/cns.13750)

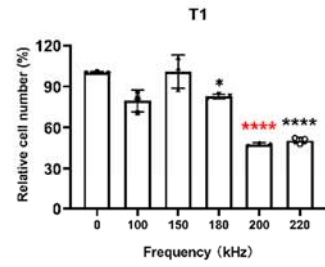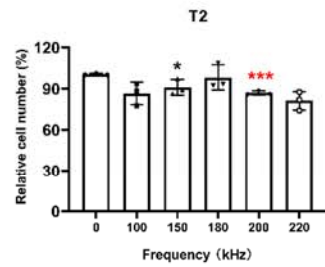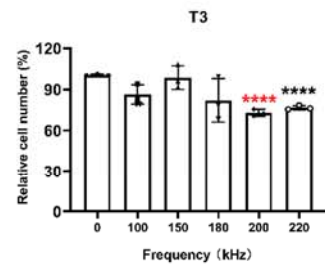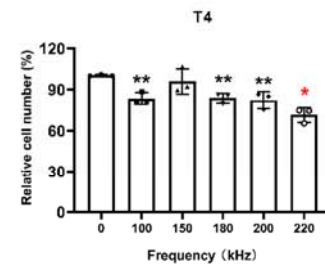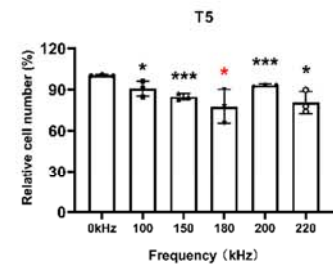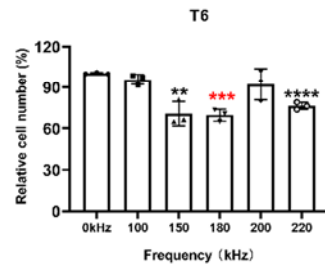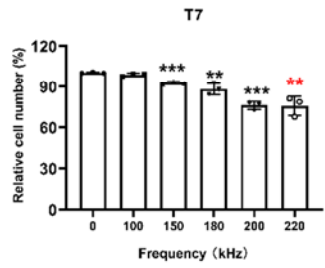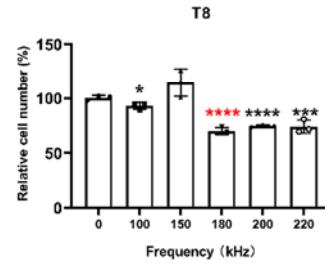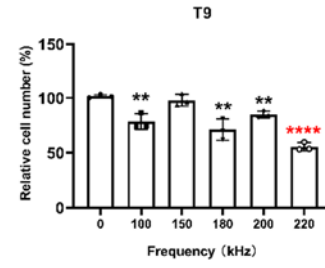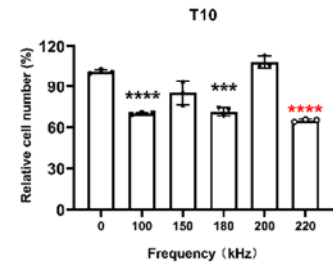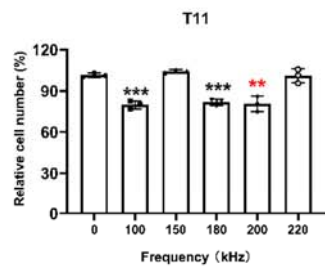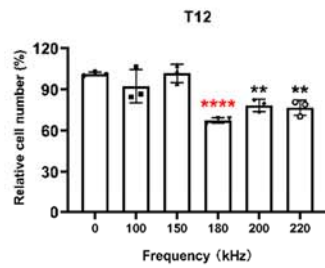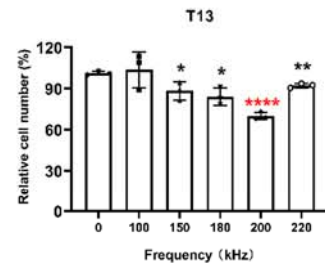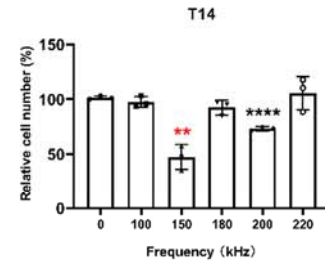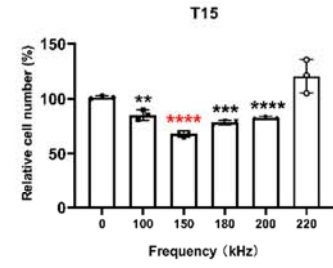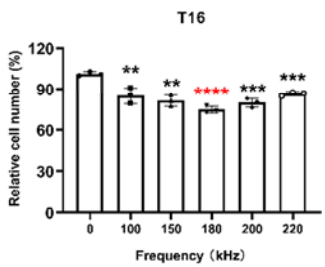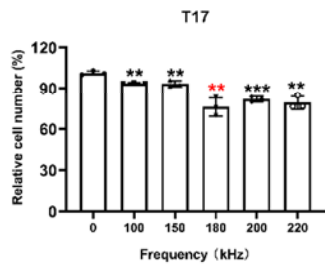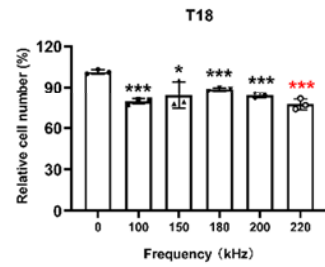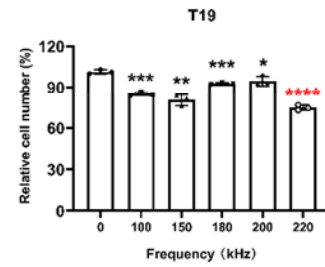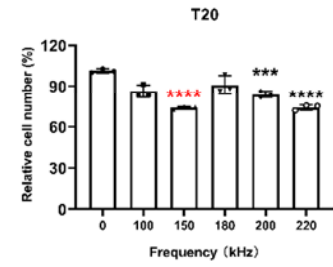

Supplement: Supplementary file 1 — Fig S1 [file CNS-27-1587-s003.pdf]

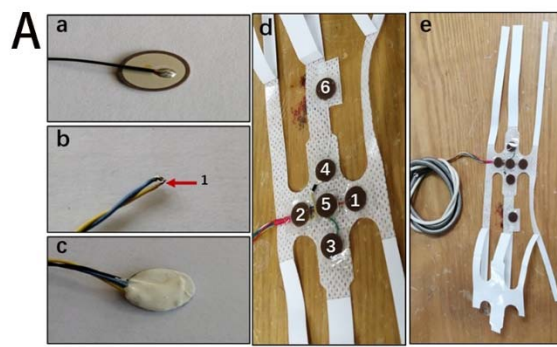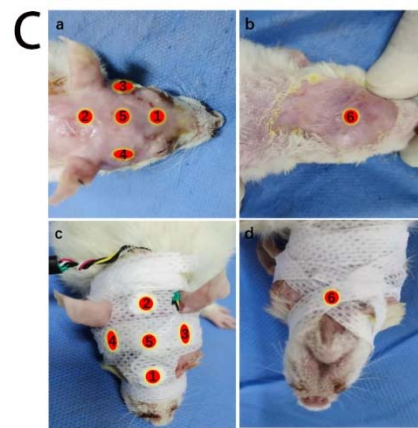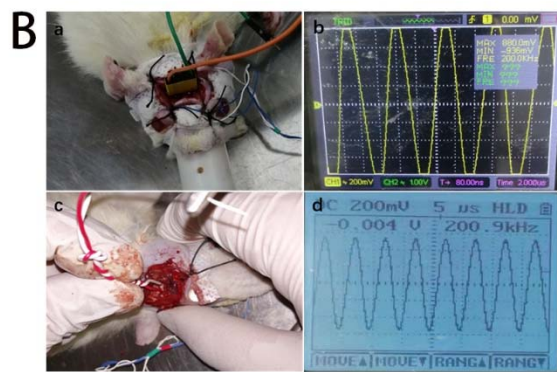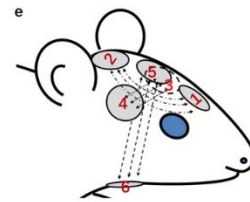

Supplement: Supplementary file 2 — Fig S2 [file CNS-27-1587-s002.pdf]

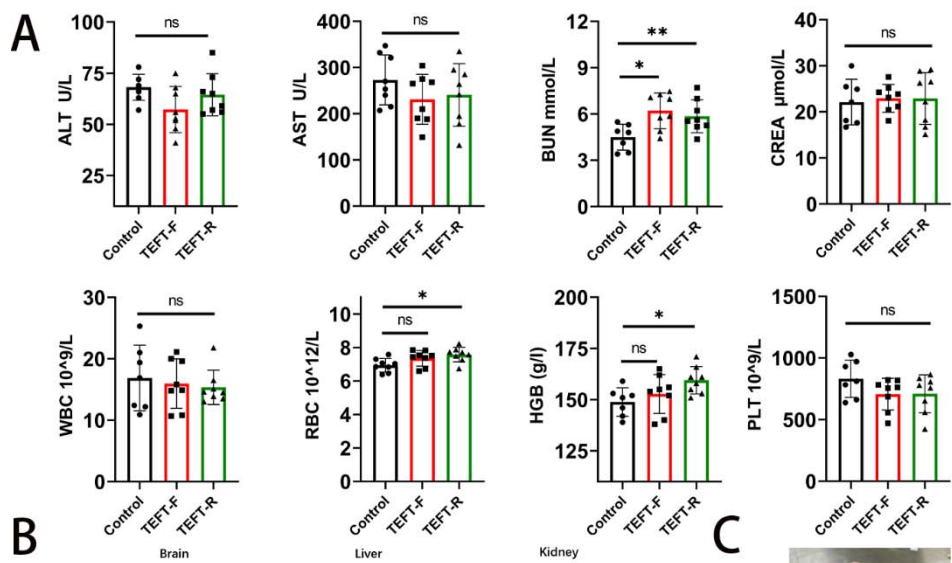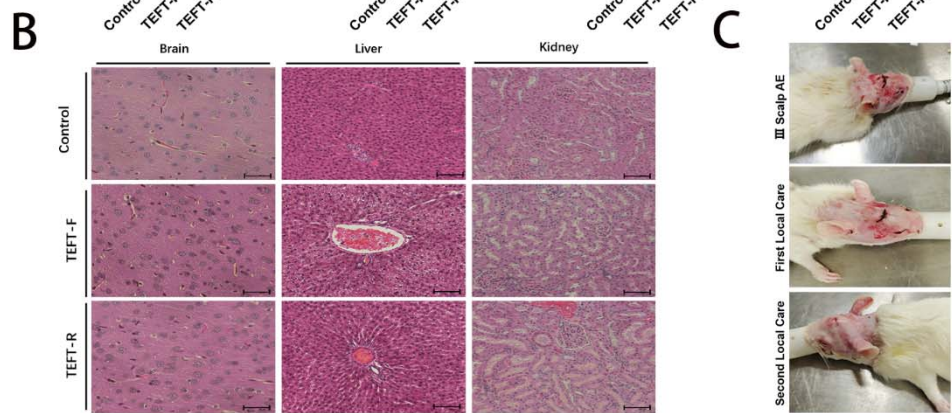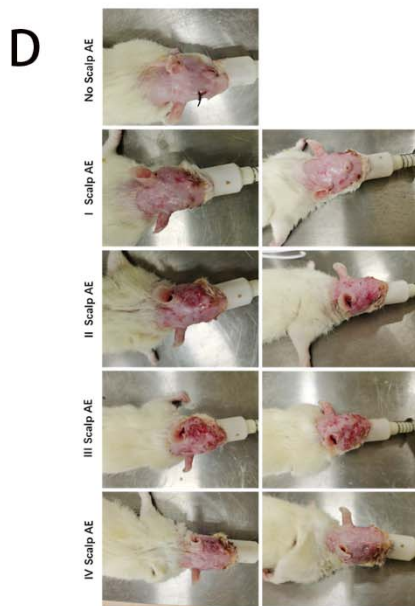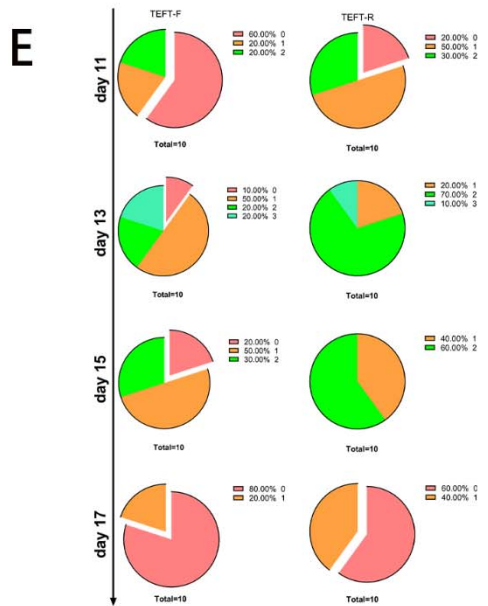

Supplement: Supplementary file 3 — Fig S3 [file CNS-27-1587-s001.pdf]
